# Microbial composition analyses by 16S rRNA sequencing: A proof of concept approach to provenance determination of archaeological ochre

**DOI:** 10.1371/journal.pone.0185252

**Published:** 2017-10-18

**Authors:** Claire E. Lenehan, Shanan S. Tobe, Renee J. Smith, Rachel S. Popelka-Filcoff

**Affiliations:** 1 College of Science and Engineering, Flinders University, Adelaide, South Australia, Australia; 2 Chemistry and Physics, Arcadia University, Glenside, PA United States of America; 3 College of Medicine and Public Health, Flinders University, Adelaide, South Australia, Australia; Seoul National University College of Medicine, REPUBLIC OF KOREA

## Abstract

Many archaeological science studies use the concept of “provenance”, where the origins of cultural material can be determined through physical or chemical properties that relate back to the origins of the material. Recent studies using DNA profiling of bacteria have been used for the forensic determination of soils, towards determination of geographic origin. This manuscript presents a novel approach to the provenance of archaeological minerals and related materials through the use of 16S rRNA sequencing analysis of microbial DNA. Through the microbial DNA characterization from ochre and multivariate statistics, we have demonstrated the clear discrimination between four distinct Australian cultural ochre sites.

## Introduction

A fundamental question in archaeological studies is the concept of “provenance”, where the origins of a material or artefact can be characterized and determined [1]. Ascertaining provenance can provide information on the object’s movement from the original source due to cultural exchange. Analytical methods capable of determining inherent characteristics of the original deposit or source, give valuable information that can assist with source attribution and in reconstructing past exchange. Ochre is a complex mineral pigment frequently observed in archaeological sites worldwide, and is often of unknown provenance. The term “ochre” is broadly used to describe natural iron oxide based pigments that range in color from red to purple, as well as yellow to brown, depending on the type and amount of iron oxide present and the mixture of other minerals and organic materials [2–7]. The major iron oxides present are hematite [Fe_2_O_3_] and goethite [FeO(OH)_x_ or α-FeOOH]. It is well known that ochres were widely traded in the Australian Aboriginal context, and recent studies have demonstrated trace elemental analysis by neutron activation analysis (NAA) of ochre for determining this “trace elemental fingerprint” with a view for provenancing ochre samples [8–13]. In addition to these studies, researchers have also used techniques such as various forms of mass spectrometry, X-ray diffraction and quartz-isotope ratios, especially for Australian ochre [14–17], Elemental and mineralogical profiling have been established as a viable methodology for provenance studies of complex mineralogical samples. However, to date no research to date has examined the possibility of the use of soil microbiology to provenance complex archaeological minerals. These novel 16S rRNA sequencing methods offer potential to understand the microbial “fingerprint” of a source that also includes the source history. Data from 16S rRNA sequencing studies provide profiles of ochre sources that are both independent of and complementary to elemental and mineralogical analyses.

The DNA profile of microrganisms within samples is a rich source of information that can be exploited to further understand archaeological problems. DNA profiling has been used for paleoclimatic reconstruction [18] and understanding environmental change [19]. To our knowledge, DNA profiling has not been applied to archaeological provenancing. Recent research has demonstrated the utility of DNA profiling of microbial communities for forensic discrimination (provenance) of visually similar soils [20, 21]. DNA profiles from the entire genetic composition (bacteria, fungi, plant material etc.) within the soil generate a rich data set for meaningful comparison and discrimination of samples. These results can be combined with those of trace elemental analysis for further resolution [20].

In order to investigate the powerful potential of using microbial DNA profiles for archaeological provenancing, a proof of concept study utilizing the 16S rRNA profile of ochres from four well-documented Australian sites were sequenced and compared [9, 10, 16, 22, 23]. This study focuses on the provenance of ochre minerals, however this method has potential for characterising and understanding other related cultural material.

**Fig 1:**
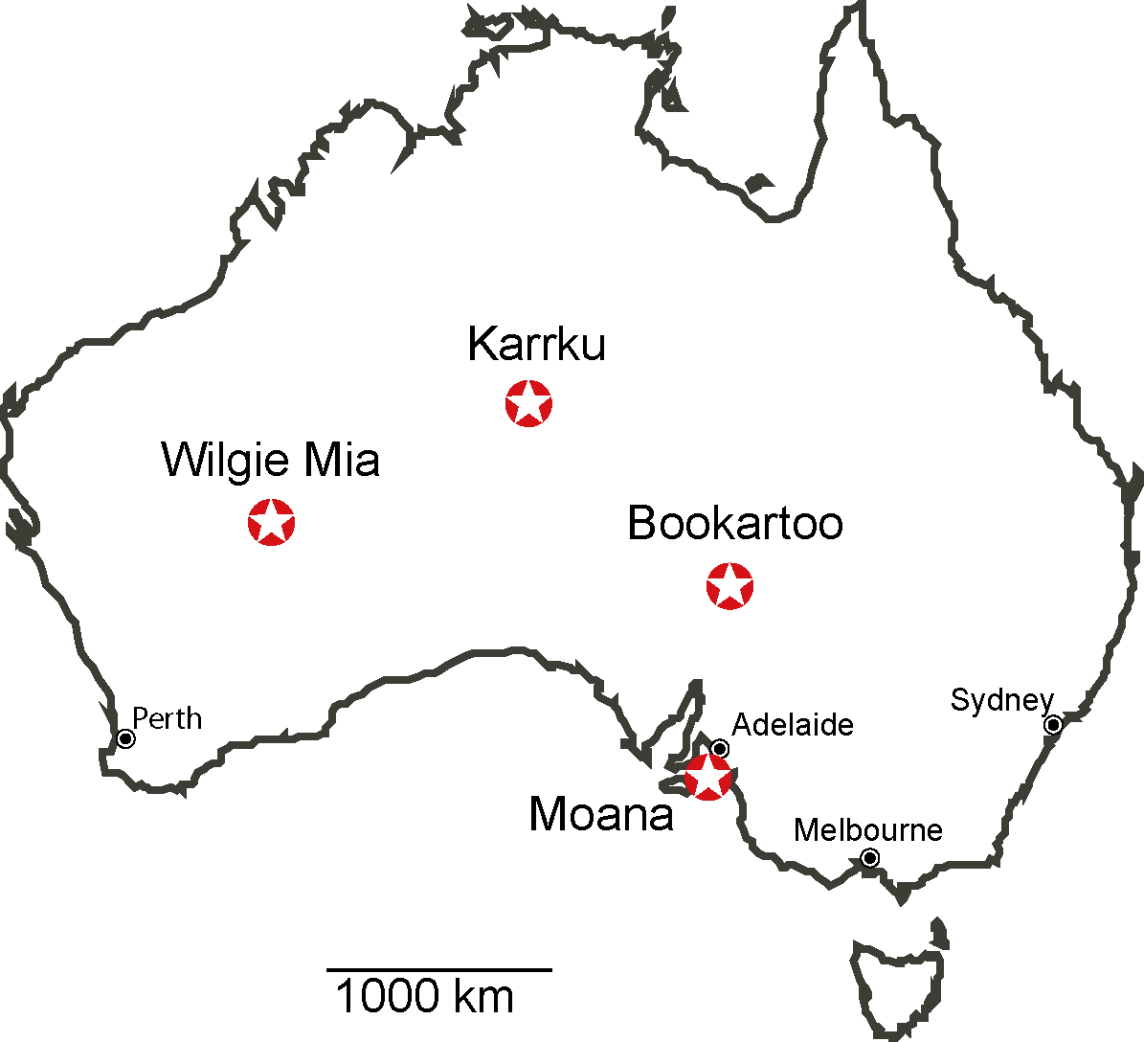
Map indicating the geographic locations of the sources sampled in this study. Modified from http://d-maps.com/carte.php?num_car=3289&lang=en.

These sites represent varying geological characteristics as well as varying site histories, both geological and cultural. (Fig 1) Wilgie Mia (sample OCH117), a weathered banded iron formation in Western Australia, is one of the most well known Australian ochre deposits due to the quality of the ochre and the extensive scale of the ochre excavations over the past thousands of years [24]. Similarly, Bookartoo (sample OCH255, Flinders Ranges, South Australia), and Karrku (sample OCH095, Northern Territory) are also banded formations of high quality ochre [25]. In contrast, Moana (sample OCH037) is an exposed coastal site in South Australia and subject to weathering.

Microbiological profiling of soils has been demonstrated to allow discrimination between different, but visually similar, soil types in forensic science as well as environmental microbial ecology [21, 26, 27]. The factors that contribute to bacterial community composition have been shown to include: pollutants, aquatic or terrestrial substrate, and environmental factors [28–32]. Based on successful discrimination of soils for forensic and ecological purposes, this avenue of investigations offers an exciting possibility for archaeological provenance.

## Materials and methods

### Site description and sampling

Four samples from the four sites (Bookartoo [OCH255], Karrku [OCH095], Moana [OCH037] and Wilgie Mia [OCH117]) were selected. All samples used were obtained either through the South Australian Museum or directly from the researcher who collected the sample. In either case, samples had been packaged individually. No permits were required for the described study. The project has approval number 4670 from the Social and Behavioural Research Ethics Committee of Flinders University.

Once in the preparation laboratory, samples were handled with nitrile gloves that were changed between samples. The surface material was removed prior to preparation and the sample preparation continued with a freshly exposed area of sample [16, 22, 23].

### Sample preparation, microbial community DNA extraction and sequencing

The ochre samples were powdered in an agate mortar and pestle and dried in a desiccator. Stringent methods were used in cleaning materials between samples, which included disposable paper and plastic materials where possible; and remaining items cleaned thoroughly with pyroneg detergent, deionized water, ethanol, and dried between samples. Approximately 3 grams of each was divided into three 1-gram portions, for a total of 12 samples (3 samples times the 4 sites investigated). These samples were prepared into individual sterile glass vials for storage prior to molecular work.

DNA extraction took place in a dedicated extraction and PCR set-up laboratory with protocols in place to ensure no PCR products were present. All benches and equipment were sterilized prior to use with a 3% bleach wash followed by pure molecular grade ethanol. All reagents and consumables were sterilized with ultra-violet light before use in a crosslinker. To ensure quality control, negative samples were processed at the same time as the ochre samples and subjected to the same scientific procedures prior to sequencing.

Microbial community DNA was extracted using the PowerSoil DNA Isolation Kit (MoBio laboratories, Inc., Carlsbad, CA, USA). DNA quality and concentration were determined by 1.5% TBE agarose gel electrophoresis and by using a Qubit fluorometer (Qubit dsDNA HS Assay Kit; Life Technologies). Control samples did not show any gel product and their Qubit reading was below detection limits/extremely low. Some carry over DNA from the bacteria, from which the DNA polymerase is derived, is expected and unavoidable, so this very low signal from the negative control was within acceptable laboratory limits.

High molecular weight DNA from the ochre samples was then sent to the Molecular Research LP (MR DNA; Texas, USA) for 16S rRNA gene based sequences on the Illumina MiSeq platform using the MiSeq Reagent Kit v3 (600 cycle) (Illumina). Bacterial diversity of ochre samples was determined by amplification of the 16SS rRNA gene using the primers 27F (5’-AGRGTTTGATCMTGGCTCAG -3’) and 519R (5’-GTNTTACNGCGGCKGCTG -3’).

The Paired-End reAd merger (PEAR) v.0.9.5 [33] was used to pair forward and reverse reads from each ochre sample. Merged DNA sequences were processed using Quantitative Insights Into Microbial Ecology (QIIME) [34] v.1.8.0 and UPARSE [35] as previously described, however without the removal of singletons The quality filtering criteria were a minimum 200bp in length, minimum quality score of 30, no mismatches in the primers sequence and no more than 6 ambiguous bases. USEARCH [36] was used to perform filtering of duplicate sequences and chimera removal. The remaining sequences were clustered into operational taxonomic units (OTUs) based on sequence similarity using uclust and Greengenes database (13_08) as a references in QIIME, with a minimum identity cutoff of 97%. Sequences were rarefied to the lowest sequence number of 5199 to remove any bias based on differences in sequencing depth.

The full data set is available at the Harvard Dataverse: https://dataverse.harvard.edu/dataset.xhtml?persistentId=doi:10.7910/DVN/FUB62C

### Data analysis

Differences in overall taxonomic composition between the ochre samples were analyzed using the PERMANOVA+ version 1.0.3 3 add-on to PRIMER [37, 38]. Non-metric Multi-Dimensional scaling (NMDS) of Bray-Curtis similarities was performed as an unconstrained ordination method to graphically visualize the multivariate patterns in the taxa associated with the ochre samples.

Canonical analysis of principal coordinates (CAP) [38] on the sum of squared canonical correlations was used as a constrained ordination to determine whether there were any significant differences between microbial community compositions between the ochre samples. The *a priori* hypothesis that the taxonomic compositions between the ochre samples were different was tested in CAP by obtaining a *P-value* using 9999 permutations. CAP ordinations were generated using order level taxonomic classifications.

Where significant differences were found using CAP, the percent contribution of each taxa to the separation between ochre samples were assessed using similarity percentage (SIMPER) analysis [39]. The resulting top 90 percent of all taxa were used to determine those taxa that were driving the dissimilarity between ochre samples.

## Results/discussion

**Fig 2:**
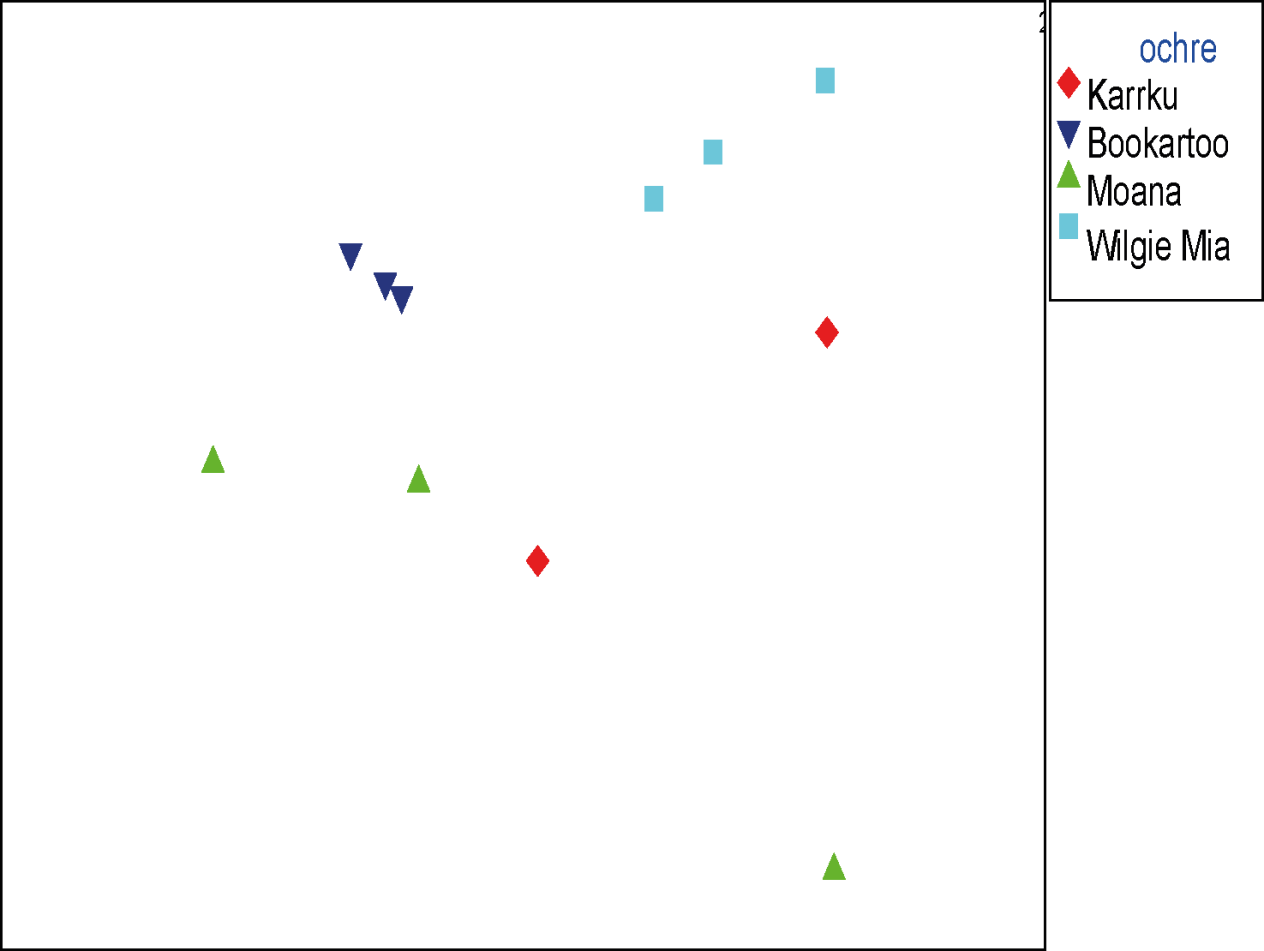
Comparison of ochre samples. NMDS ordination derived from a Bray-Curtis similarity matrix calculated from the square-root transformed abundance of 16S rRNA sequences matching the Greengenes database (13_08), order level.

**Fig 3:**
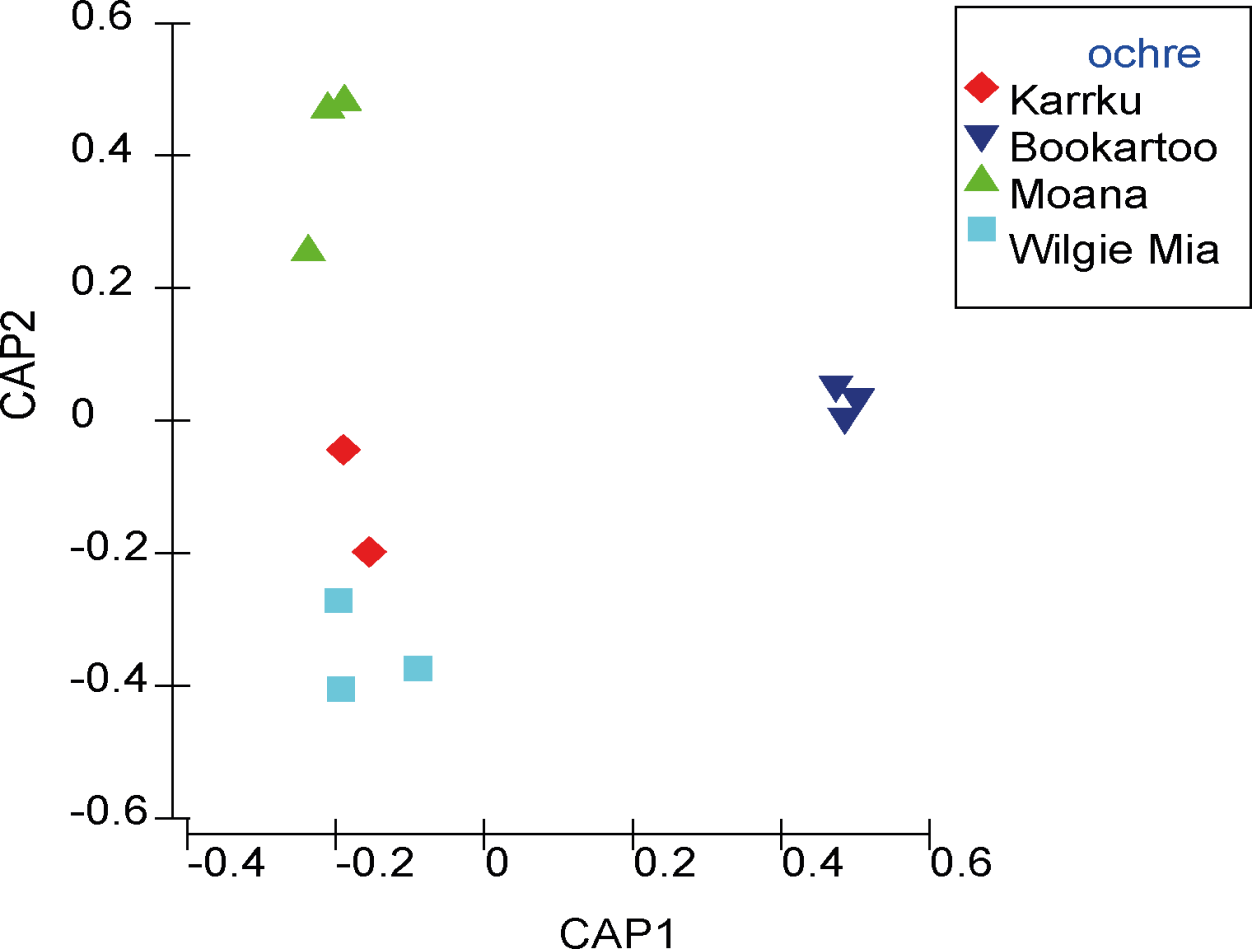
Comparison of the ochre samples. CAP analysis (using m = 4 principal coordinate axes) is derived from the sum of squared canonical correlations of 16S rRNA sequences matching the Greengenes database (13_08), order level.

All samples had data returned with the exception of one of the Karrku samples (OCH95), of which one replicate did not amplify (S1 and S2 Tables). Data were analyzed using CAP to examine the hypothesis that the ochre samples were the same, as well as the NMDS as an unconstrained ordination (no hypothesis to constrain the data). Unconstrained (NMDS) and constrained (CAP) multivariate analysis demonstrated a clear separation of data (P-value = 0.0003) between the ochre samples (Figs 2 and 3 and Tables 1 and 2).

**Table 1:** Results of CAP analysis (using m = 6 principal coordinate axes, explaining 99.8% of the total variation) testing the hypothesis that taxonomic composition differs for order level classifications associated with different ochre samples.

	Factor	m	Allocation Success % (ratio correct:misclassified)					δ^2^	*P*-value (δ^2^)	*P-*value (trace)
			Moana	Karrku	Wilgie Mia	Bookartoo	Total			
**Taxonomy**	Order	4	66.7 (2:3)	50 (1:1)	100 (3:3)	100 (3:3)	81.81	0.99	**0.02**	**0.0003**

**Table 2:** Results of CAP analysis (using m = 1 principal coordinate axes, explaining 100% of the total variation) testing the hypothesis that elemental composition differ for each ochre sample.

	Factor	m	Allocation Success % (ratio correct:misclassified)					δ^2^	*P*-value (δ^2^)	*P-*value (trace)
			Moana	Karrku	Wilgie Mia	Bookartoo	Total			
**Elemental data**	Order	6	0 (0:1)	0 (0:1)	0 (0:1)	100 (1:1)	25	1	1	1

The clear separation in statistical space between data points in the NMDS and the CAP plots indicate that the ochre samples can be readily distinguished based on taxonomic composition. In addition, the replicate samples from the ochre sources cluster very closely within the groups, further supporting the evidence of low levels of variability between replicates of the same sample [21]. Similar results have been observed in related soil types. The differences in the ochre groups can also be observed as reflecting the origins of their original sites and site genesis. For instance, while both Bookartoo and Wilgie Mia are banded iron formations, each has a distinct site history over geological time. In the case of Bookartoo, the ochre developed as the weathering of pyrite in dolomitic beds, while Wilgie Mia ochre was thought to be hematite oxidized from magnetite [14]. In contrast, Moana is from a coastal, weathered sedimentary formation [14]. Each of these sites, due to their geographic origin and subsequent genesis over geological time, will have a different microbial DNA signature [21]. Separation seen in both NMDS and CAP suggests that the data points are not simply conforming to the more hypothesis-driven CAP analysis.

**Fig 4:**
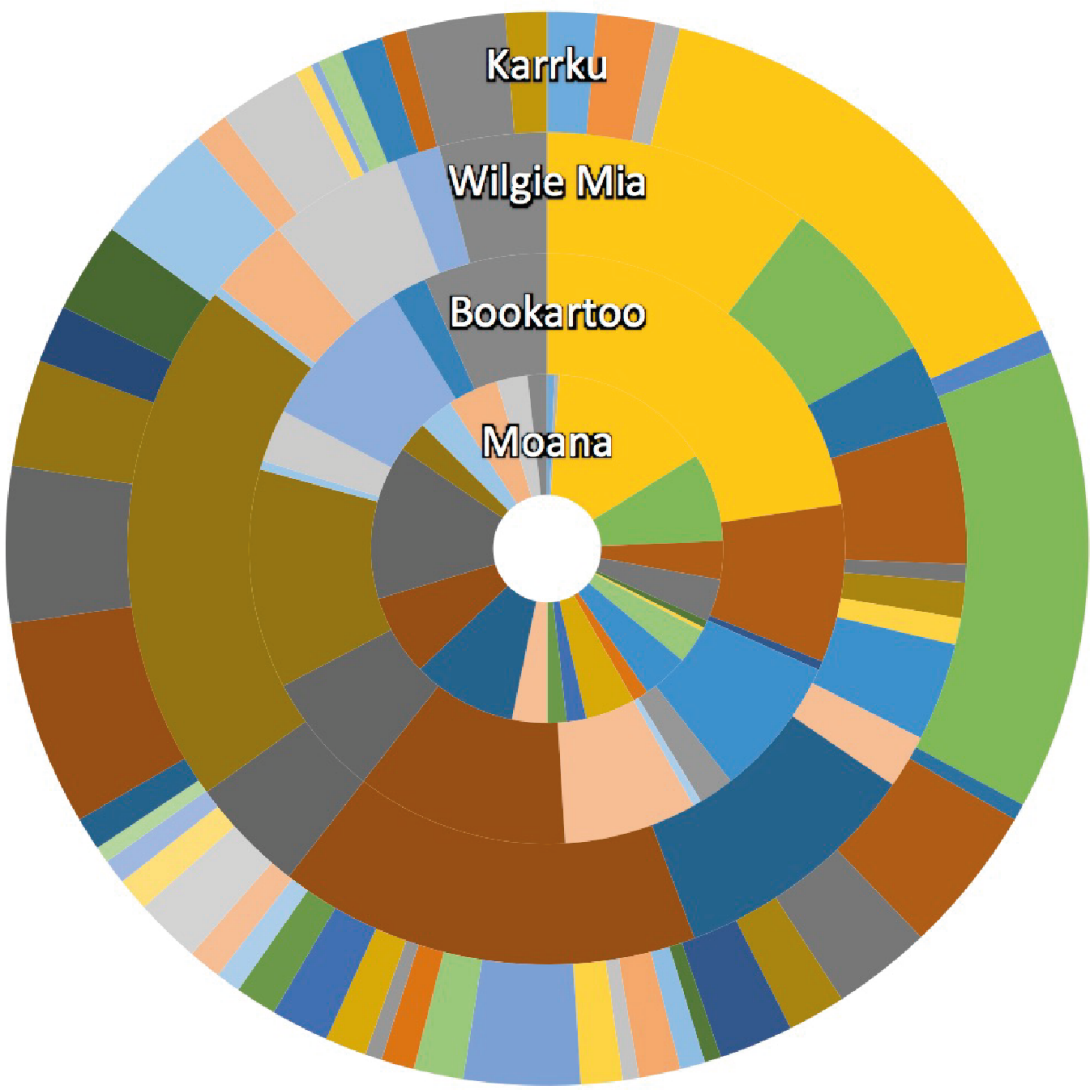
Rank abundance representing the percent contribution of order level taxonomy to the dissimilarity of the ochre samples. Only taxa that were consistently contributing (i.e. Diss/SD > 1.4) to the dissimilarity have been included. Cut off percentage is 90%. Percent contributions were calculated based on square root transformed relative abundance values. The legend can be found in the SI.

SIMPER analysis supports the separation of data with clear shifts in the taxonomic composition between ochre samples (Fig 4 and S1 Fig).

Previous analysis shows that environmental factors are a major contributor to the composition of microbial communities [30, 32, 40]. Here, SIMPER revealed 46 taxa that were driving the dissimilarity between ochre samples, demonstrating a powerful discriminatory method for separating ochre samples. Differences in environmental conditions could influence the microbial communities structure for each of the four sites investigated [32].

## Conclusion

This study demonstrates the novel application of 16S rRNA sequencing analysis of microbial DNA towards identifying the provenance of archaeological samples, in this case Indigenous ochre samples from four locations. The use of the microbial data and statistical analysis reveals that despite time and sample movement away from its origins, microbial information in a given soil or geological sample can be used as a “signature” for the original source of the material. The 16S rRNA sequencing data in conjunction with elemental data has the potential to enhance provenance determination for complex archaeological samples such as ochre and soils. More case studies can be examined to evaluate larger data sets and samples from archaeological contexts, including multiple samples from the same/differing site and strata, however we have demonstrated that trace microbial content in archaeological samples provide a yet unexploited source of information for archaeological provenance studies.

## Supporting information

S1 Fig Plot showing the relative abundance in each sample of 70 different orders. (PDF)

S1 Table Number of sequences obtained for each ochre sample. (PDF)

S2 Table Order level relative proportion of matches to the Greengenes (13_08) database. (PDF)
