# Supplementary material for: Microbial composition analyses by 16S rRNA sequencing: A proof of concept approach to provenance determination of archaeological ochre
Source: PLoS One. 2017 Oct 18;12(10):e0185252. doi: 10.1371/journal.pone.0185252 (PMC5646784; doi:10.1371/journal.pone.0185252)
Supplement: S1 Fig — (PDF) [file pone.0185252.s001.pdf]

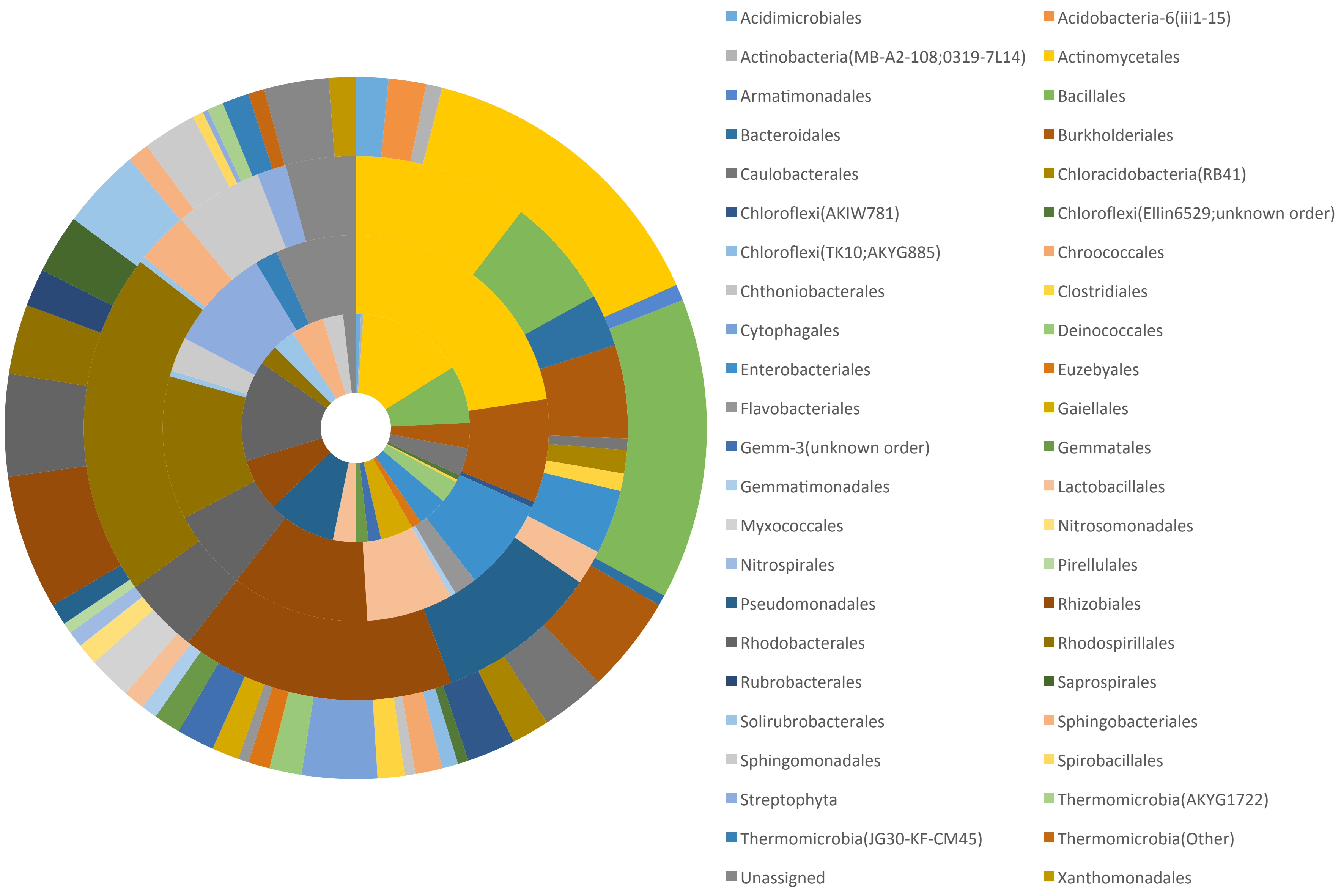
Acidimicrobiales
Acidobacteria-6(iii1-15)
Actinobacteria(MB-A2-108;0319-7L14)
Actinomycetales
Armatimonadales
Bacillales
Bacteroidales
Burkholderiales
Caulobacterales
Chloracidobacteria(RB41)
Chloroflexi(AKIW781)
Chloroflexi(Ellin6529;unknown order)
Chloroflexi(TK10;AKYG885)
Chroococcales
Chthoniobacterales
Clostridiales
Cytophagales
Deinococcales
Enterobacteriales
Euzebyales
Flavobacteriales
Gaiellales
Gemm-3(unknown order)
Gemmatales
Gemmatimonadales
Lactobacillales
Myxococcales
Nitrosomonadales
Nitrospirales
Pirellulales
Pseudomonadales
Rhizobiales
Rhodobacterales
Rhodospirillales
Rubrobacterales
Saprospirales
Solirubrobacterales
Sphingobacteriales
Sphingomonadales
Spirobacillales
Streptophyta
Thermomicrobia(AKYG1722)
Thermomicrobia(JG30-KF-CM45)
Thermomicrobia(Other)
Unassigned
Xanthomonadales
