# Supplementary material for: Microbial composition analyses by 16S rRNA sequencing: A proof of concept approach to provenance determination of archaeological ochre
Source: PLoS One. 2017 Oct 18;12(10):e0185252. doi: 10.1371/journal.pone.0185252 (PMC5646784; doi:10.1371/journal.pone.0185252)
Supplement: S1 Table — (PDF) [file pone.0185252.s002.pdf]

Table S1 – Number of sequences obtained for each ochre sample

Sample	# sequences
Wilgie Mia R1	5199
Moana R1	11217
Karrku R1	18726
Karrku R2	19992
Wilgia Mia R2	23245
Moana R2	29130
Wilgia Mia R3	29159
Moana R3	59429
Bookartoo R1	60765
Bookartoo R2	64277
Bookartoo R3	65972
