# Supplementary material for: Microbial composition analyses by 16S rRNA sequencing: A proof of concept approach to provenance determination of archaeological ochre
Source: PLoS One. 2017 Oct 18;12(10):e0185252. doi: 10.1371/journal.pone.0185252 (PMC5646784; doi:10.1371/journal.pone.0185252)
Supplement: S2 Table — (PDF) [file pone.0185252.s003.pdf]

Table S2 – Order level relative proportion of matches to the Greengenes (13_08) database

**Taxon**	Moana R3	Moana R2	Bookartoo R2	Bookartoo R3	Wilgia Mia R3	Karrku R2	Bookartoo R1	Karrku R1	Wilgia Mia R2	Moana R1	Wilgie Mia R1
**Unassigned**	0.006539719	0.002500481	0.019619158	0.018657434	0.000577034	0.032890941	0.008270821	0.008078477	0.013079438	0.000192345	0.043854587
**Acidobacteria-6 (o__iii1-15)**	0.000192345	0.111367571	0.004808617	0.004231583	0	0	0.004423928	0	0	0.000192345	0.000192345
**Sva0725**	0	0	0.000192345	0	0	0	0	0	0	0	0
**Chloracidobacteria (PK29)**	0	0	0	0	0	0	0.000384689	0	0	0	0
**Chloracidobacteria (RB41)**	0	0	0.009424889	0.007693787	0.012502404	0	0.000384689	0	0	0	0
**DS-18**	0	0	0.000577034	0.000384689	0	0	0	0	0	0	0
**Acidimicrobiales**	0.001923447	0.000192345	0.006347374	0.004423928	0.000192345	0	0.001923447	0	0	0	0
**Actinomycetales**	0.255049048	0.152529333	0.334295057	0.382573572	0.046932102	0.417964993	0.304289286	0.083092902	0.164839392	0.125024043	0.070013464
**Micrococcales**	0	0	0.000769379	0.000577034	0	0	0	0	0	0	0
**Coriobacteriales**	0	0.000384689	0	0	0	0	0	0	0	0	0
**MB-A2-108 (0319-7L14)**	0.000577034	0	0.001923447	0.001538757	0	0	0.000384689	0	0	0	0

**Euzebyales**	0.012694749	0	0.003654549	0.00288517	0.000192345	0	0.000192345	0	0	0	0
**Rubrobacterales**	0.000192345	0	0.009232545	0.00326986	0	0	0.004231583	0	0	0	0
**Gaiellales**	0.022311983	0.055395268	0.002692826	0.002500481	0	0	0.001346413	0	0	0	0
**Solirubrobacterales**	0.044431621	0.003654549	0.029236392	0.025774187	0.000577034	0	0.011733026	0.000384689	0	0	0.000192345
**Armatimonadetes0319-6E2 (unknown order)**	0	0	0	0	0	0	0.000384689	0	0	0	0
**Armatimonadales**	0	0	0.002115791	0.000192345	0	0	0.001154068	0	0	0.000192345	0
**ArmatimonadetesOPB50 (unknown order)**	0	0	0.000192345	0	0	0	0	0	0	0	0
**Bacteroidales**	0.009424889	0	0.000192345	0.000192345	0	0	0.000577034	0	0.008463166	0	0.028659358
**Cytophagales**	0.021157915	0.009617234	0.018657434	0.010578957	0	0	0.028082324	0	0	0	0
**Flavobacteriales**	0.001154068	0	0	0.000577034	0	0.000192345	0.000961723	0.003654549	0.000769379	0	0.000192345
**Sphingobacteriales**	0	0.044816311	0.002500481	0.001154068	0.016541643	0	0.001154068	0	0	0.028467013	0.02135026
**Rhodothermales**	0	0	0	0.0001923	0	0	0.0005770	0	0	0	0

				45			34				
**Saprospirales**	0	0	0.017695711	0.012117715	0	0	0.008463166	0	0	0	0
**Chloroflexi (unknown order)**	0	0	0.000384689	0.000384689	0	0	0	0	0	0	0
**Ardenscatenales**	0	0	0.000577034	0.000577034	0	0	0	0	0	0	0
**Caldilineales**	0	0	0	0.000577034	0	0	0	0	0	0	0
**SBR1031**	0	0	0	0	0.000577034	0	0	0	0	0	0
**AKIW781**	0	0	0.013079438	0.012887094	0.000192345	0	0.001731102	0.000192345	0	0	0
**Chloroflexales**	0	0	0.000192345	0.000577034	0	0	0	0	0	0	0
**Herpetosiphonales**	0	0	0.000192345	0	0	0	0	0	0	0	0
**Roseiflexales**	0	0	0	0.000384689	0	0	0	0	0	0	0
**Ellin6529 (unknown order)**	0	0.004616272	0.000769379	0.000192345	0.000192345	0	0.000577034	0	0	0	0
**Gitt-GS-136 (unknown order)**	0.004039238	0	0.000577034	0.000192345	0	0	0	0	0	0	0
**Ktedonobacteria (unknown order)**	0	0	0.000384689	0	0	0	0	0	0	0	0

**S085 (unknown order)**	0	0	0	0.000192345	0	0	0	0	0	0	0
**TK10 (unknown order)**	0	0	0.000192345	0	0	0	0	0	0	0	0
**TK10 (AKYG885)**	0	0	0.001538757	0.001346413	0	0	0.000577034	0	0	0	0.000192345
**TK10 (B07_WMSP1)**	0	0	0.000192345	0.000192345	0	0	0	0	0	0	0
**Thermomicrobia (Other)**	0	0	0.000961723	0.000577034	0	0	0.000769379	0	0	0	0
**Thermomicrobia (unknown order)**	0	0	0.000961723	0.000384689	0	0	0	0	0	0	0
**Thermomicrobia (AKYG1722)**	0	0	0.001923447	0.001346413	0	0	0.000192345	0	0	0	0
**Thermomicrobia (JG30-KF-CM45)**	0	0	0.002692826	0.002692826	0	0	0.001346413	0.006732064	0	0	0
**Thermobaculales**	0	0	0.000192345	0.000192345	0	0	0.000769379	0	0	0	0
**Cyanobacteria (unknown order)**	0	0	0.000192345	0	0	0	0	0	0	0	0
**Chlorophyta**	0	0	0.000192345	0	0	0	0	0	0	0	0
**Streptophyta**	0.000384689	0.239276784	0	0.000384689	0.003077515	0.025774187	0.000192345	0.039238315	0	0.024812464	0.008847855
**ML635J-21**	0	0	0.0001923	0	0	0	0	0	0	0	0

**(unknown order)**			45								
**Nostocales**	0	0	0	0.000192345	0	0	0	0	0	0	0
**Chroococcales**	0	0	0.000961723	0.004616272	0	0	0.002308136	0	0	0	0
**FBP (unknown order)**	0	0	0.000769379	0.000769379	0.014041162	0	0	0	0	0.011155992	0
**Bacillales**	0.037507213	0.038276592	0.239084439	0.283131371	0.019042123	0.028082324	0.417964993	0.268897865	0.008655511	0.099634545	0.10886709
**Gemellales**	0	0	0	0	0	0.249471052	0	0	0.000961723	0	0
**Lactobacillales**	0.014041162	0	0.003654549	0.000192345	0.010963647	0.001154068	0.003077515	0.0723216	0.004616272	0.027697634	0.000192345
**Clostridia (unknown order)**	0	0	0	0.000192345	0	0	0	0.04847086	0	0	0
**Clostridiales**	0	0	0.002115791	0.001538757	0	0	0.003462204	0	0.010194268	0.001154068	0
**Gemm-1 (unknown order)**	0	0	0.000192345	0	0	0	0	0	0	0	0
**Gemm-3 (unknown order)**	0	0	0.004808617	0.005000962	0	0	0.005385651	0	0	0.020003847	0
**Gemm-5 (unknown order)**	0	0	0.000192345	0	0	0	0.000192345	0	0	0.00288517	0
**Gemmatimonadetes (Other)**	0	0	0.000384689	0.000384689	0	0	0.000192345	0	0	0	0

**Gemmatimonadetes (unknown order)**	0	0	0.001731102	0.001731102	0	0	0	0	0	0	0
**Gemmatimonadales**	0	0	0.001731102	0.001538757	0	0	0.000192345	0.000192345	0	0	0
**Nitrospirales**	0	0	0.000961723	0.001346413	0	0	0.000384689	0	0	0	0
**WD2101**	0	0	0.000577034	0.000384689	0	0	0.000384689	0	0	0	0
**Gemmatales**	0.000192345	0.019042123	0.005193306	0.003077515	0	0	0.000192345	0	0	0	0
**Pirellulales**	0.011155992	0	0.001346413	0.000384689	0	0	0.000192345	0	0	0	0
**Planctomycetales**	0.012887094	0	0.000384689	0	0	0	0	0	0	0	0
**Caulobacterales**	0.110021158	0	0.017311021	0.016541643	0.00326986	0	0.012502404	0	0	0	0.000192345
**Rhizobiales**	0.006732064	0.043854587	0.086747451	0.085593383	0.216772456	0.095402962	0.027889979	0.031736872	0.173302558	0.109636469	0.278515099
**Rhodobacterales**	0.358722831	0.043277553	0.065974226	0.03327563	0.100211579	0.060780919	0.020003847	0.001346413	0.000384689	0.149451818	0.001923447
**Rhodospirillales**	0.013271783	0.006924409	0.020196192	0.018465089	0.408155415	0.075783805	0.015387575	0.046739758	0.472013849	0.000961723	0.193691094
**Rickettsiales**	0	0.092133103	0.000192345	0.000192345	0	0	0.000192345	0	0	0.024812464	0
**Sphingomonadales**	0.0248124	0.0003846	0.0115406	0.0069244	0.0642431	0	0.0109636	0.0165416	0.0011540	0.0057703	0.0251971

	64	89	81	09	24		47	43	68	4	53
**Betaproteobacteria (Other)**	0	0	0.000384689	0	0	0	0	0	0	0	0
**Betaproteobacteria (unknown order)**	0	0	0.000192345	0.000769379	0	0	0	0	0	0	0
**Burkholderiales**	0.001538757	0.025389498	0.022889017	0.017118677	0.037699558	0	0.068667051	0.128293903	0.001538757	0.009424889	0.054433545
**MWH-UniP1**	0	0	0	0.000192345	0	0	0	0	0	0	0
**Neisseriales**	0.002115791	0.000192345	0.000192345	0	0.006539719	0	0.000384689	0.092710137	0	0.058280439	0.000192345
**Nitrosomonadales**	0	0	0.002308136	0.001731102	0	0	0.000961723	0	0	0	0
**Deltaproteobacteria (MIZ46)**	0	0.005385651	0.000192345	0.000192345	0	0	0	0	0	0	0
**Myxococcales**	0	0	0.008270821	0.004231583	0.000192345	0	0.005385651	0	0	0	0
**Spirobacillales**	0	0	0.000577034	0.000384689	0	0	0.000577034	0	0	0	0
**Syntrophobacterales**	0	0	0.000192345	0	0	0	0	0	0	0	0
**Campylobacterales**	0	0	0	0.000192345	0	0	0	0	0	0.000384689	0
**Gammaproteobacteria (Other)**	0	0	0	0	0	0	0	0	0.000384689	0.000384689	0

**Aeromonadales**	0	0	0	0	0.001538757	0	0	0	0.000192345	0	0
**Alteromonadales**	0	0	0.000384689	0.000577034	0	0	0.000192345	0	0.013079438	0	0
**Chromatiales**	0	0	0	0.000192345	0	0	0	0	0	0	0
**Enterobacteriales**	0	0.000384689	0.000192345	0	0.008463166	0.004808617	0	0.065204847	0.040007694	0.111559915	0.000769379
**Pasteurellales**	0	0	0	0	0	0.002500481	0	0	0	0	0
**Pseudomonadales**	0.013464128	0.064435468	0.000192345	0.001923447	0.023658396	0.005193306	0.004039238	0.085785728	0.086362762	0.187920754	0.162531256
**Thiotrichales**	0	0	0.000192345	0	0	0	0	0	0	0	0
**Vibrionales**	0	0	0	0	0.002115791	0	0	0	0	0	0
**Xanthomonadales**	0	0	0.005577996	0.002692826	0	0	0.001154068	0	0	0	0
**Synergistales**	0.004423928	0	0	0	0	0	0	0	0	0	0
**TM7SC3 (unknown order)**	0	0	0	0.000192345	0	0	0	0	0	0	0
**TM7TM7-1 (unknown order)**	0	0.005000962	0	0	0	0	0	0	0	0	0
**TM7TM7-3 (Other)**	0	0	0	0	0.0005770	0	0	0	0	0	0

					34						
**TM7TM7-3 (unknown order)**	0	0.0038468 94	0	0	0	0	0	0	0	0	0
**Chthoniobacterales**	0	0	0.0009617 23	0.0001923 45	0	0	0.0005770 34	0	0	0	0
**Deinococcales**	0.0090402	0.0271206	0.0017311 02	0.0013464 13	0.0009617 23	0	0.0121177 15	0.0003846 89	0	0	0
